# Trace elements and organic contaminants in tissues of minke whale (*Balaenoptera acutorotstrata*) and its feed from Icelandic waters

**DOI:** 10.1186/1751-0147-54-S1-S10

**Published:** 2012-02-24

**Authors:** Guðjón Atli Auðunsson, Gísli A Víkingsson

**Affiliations:** 1 Innovation Center Iceland, Department of Analytical Chemistry, Keldnaholti, 112 Reykjavik, Iceland; 2 Marine Research Institute Iceland, Depatment of Whale Research, Skúlagata 4, 101 Reykjavik, Iceland

## 

In a research project by Iceland under the auspice of the Marine Research Institute Iceland in 2003-2007, 200 minke whales (*Balaenoptera acutorostrata*) were caught in Icelandic waters. Within this research effort of Iceland, a comprehensive research activites were devoted to analysis of trace elements and organic contaminants in both different tissues of the minke whale and its possible prey species as revealed by stomach content. The scientific questions asked by these studies were the following:

1. What are the characteristics of the Icelandic minke whale stock as regards environmental contaminants compared to other stocks of minke whale worldwide?

2. Can inorganic and organic contaminants provide answers regarding the prey species of the minke whales?

3. Is it possible to estimate the feed consumption of the minke whale by way of contaminants in its tissues and its prey species?

4. Do biopsies provide information on the level and behaviour of contaminants in the organs of the minke whale?

5. Are the levels of environmental contaminants in tissues of minke whales detrimental to the health of the animals?

6. Do the levels of contaminants restrict the human consumption of minke whale products?

For this purpose the trace elements Hg, Cd, Zn, Cu, Pb, Se, As, Ni, Cr, and Mn were analysed in skin, muscle, liver, kidneys, ovaries and testes of 25 minke whales selected with respect to size, sex, and location around Iceland. PCBs, DDTs, HCB, HCHs, toxaphenes, chlordanes, dieldrin and PBDEs were analysed in blubber (biopsy and cross section), muscle, and liver while dioxins and dioxinlike PCBs were analysed in the ventral grooves of five animals. All these contaminants were also analysed in 50 samples of probable prey species: whole animals of cod, haddock, pollock, herring, capelin, sandeel, and krill.

The results are described and discussed with respect to the scientific objectives above with due consideration of the biological factors that may affect the levels, *e.g.* age, sex, nutritional status and trophic level (δ^15^N and δ^13^C).

